# Impact of cholangitis on survival of patients with malignant biliary obstruction treated with percutaneous transhepatic biliary drainage

**DOI:** 10.1186/s12876-023-02704-8

**Published:** 2023-03-27

**Authors:** Jarmo Niemelä, Raija Kallio, Pasi Ohtonen, Juha Saarnio, Hannu Syrjälä

**Affiliations:** 1 Department of Surgery and Medical Research Center Oulu, Oulu University Hospital and University of Oulu, Oulu, Finland; 2 Department of Oncology, Oulu University Hospital, Oulu, Finland; 3 Divisions of Operative Care and Medical Research Center Oulu, Oulu University Hospital and University of Oulu, Oulu, Finland; 4 Department of Infection Control, Oulu University Hospital, Oulu, Finland

**Keywords:** Cancer, Biliary obstruction, Percutaneous biliary drainage, Cholangitis Survival

## Abstract

**Purpose:**

To evaluate the impact of cholangitis on survival of patients with gastrointestinal cancer and malignant biliary obstruction treated with percutaneous transhepatic biliary drainage (PTBD).

**Methods:**

A retrospective registry study was performed at a tertiary center from 2000 to 2016 in Northern Finland.

**Results:**

The study included 588 patients, 258 (43.9%) patients with pancreatic cancer, 222 (37.7%) with biliary tract cancer, and 108 (18.4%) with metastasis from gastrointestinal cancers. Patient mean age was 70 years, range 26 − 93 years. There were 288 [49.0%] women. The 30-day mortality rate was 30.8% for 156 patients with cholangitis before PTBD, 19.5% for 215 patients with cholangitis after PTBD and 25.8% for 217 patients without cholangitis (*P* = 0.039). The median survival was 1.8 months for patients with cholangitis before PTBD, 3.0 months for patients with cholangitis after PTBD, and 3.2 months for patients without cholangitis (*P* = 0.002). The hazard ratio (HR) for 1-year mortality for patients with cholangitis before PTBD was 1.3 (95% CI 1.06 − 1.67, *P* = 0.015) compared to patients with cholangitis after PTBD. After successful PTBD, 54 out of 291 patients received chemotherapy; the median survival was 5.2 months with cholangitis before PTBD, 9.4 months with cholangitis after PTBD and 15.3 months without cholangitis.

**Conclusion:**

In gastrointestinal cancers with malignant biliary obstruction, survival is poorer if cholangitis occurs before PTBD compared to cholangitis after PTBD. An oncologist’s consultation is essential for assessing the possibility of chemotherapy in successfully treated PTBD patients, because of the notable survival benefit.

## Introduction

Malignant biliary obstruction is a common clinical problem typically arising from pancreatic and biliary tract cancers as well as advanced stages of other gastrointestinal cancers [1–5]. Treatment of these patients is challenging due to heterogeneity of the patient groups. Patients are usually elderly with problems associated with advanced stages of their malignancy, as well as co-morbidities and impaired general health. In our retrospective series of 643 cancer patients with malignant biliary obstruction treated with percutaneous transhepatic biliary drainage (PTBD), the median overall survival was 2.6 months [5]. Successful PTBD with a decreasing bilirubin level allows chemotherapy with several months of survival benefit compared to patients receiving only best supportive care [6, 7].

The primary treatment choice for the drainage of biliary obstruction worldwide is endoscopic retrograde cholangiopancreatography (ERCP). The available data based on a recent systematic review and network meta-analysis showed that differences between ERCP, endoscopic ultrasound-guided biliary drainage (EUS-BD) and PTBD were likely small and did not favor any of the abovementioned procedures; however, ERCP with or without endoscopic ultrasound (EUS) should be considered as the preferred modality allowing simultaneous tissue acquisition to confirm diagnoses [8]. Cholangioscopy along with EUS are reasonable methods after inconclusive ERCP-guided tissue sampling in the evaluation of indeterminate biliary strictures [9]. When standard ERCP cannulation fails, advanced ERCP cannulation techniques, percutaneous biliary drainage and EUS-BD-guided rendezvous techniques are available rescue methods for difficult bile duct access [10]. PTBD is often a suitable treatment option due to duodenal obstruction, after previous surgery that has altered patient anatomy or when endoscopic drainage has been insufficient or failed. PTBD is an invasive procedure with a risk of complications, including bleeding, bile leakage, and infections such as cholangitis and sepsis [11, 12].

There are controversial results concerning the impact of cholangitis on survival [13, 14]. The aim of our study was to explore whether cholangitis has any effects on survival in gastrointestinal cancer patients with malignant biliary obstruction treated with PTBD, compared to PTBD patients without cholangitis. A further research aim was to determine whether there were differences in survival of patients with cholangitis before or after PTBD.

## Materials and methods

### Patients

This retrospective study is a sub-analysis of our earlier study of malignant biliary obstruction treated with PTBD at a tertiary level university hospital [5]. This study focuses on the impact of cholangitis on patient survival and includes all consecutive patients with gastrointestinal cancer and malignant biliary obstruction between 2000 and 2016. PTBD procedures were performed under anesthesia by an experienced interventional radiologists using previously documented methods, guided by ultrasonography and fluoroscopy [15]. When managing cholangitis after PTBD, catheters were changed or upgraded to self-expanding metal stents or plastic stents if biliary drainage was found to be insufficient.

The patient population was divided into three groups: patients with cholangitis before or after biliary drainage and patients without cholangitis. The diagnosis and severity of cholangitis was determined according to the Tokyo Guidelines 2013 (TG13) and the severity was graded as mild, moderate, or severe [16].

## Data collection

Full electronic medical records were available for the time period, and the following data were retrospectively retrieved: age, gender, pre-procedure American Society of Anesthesiologists physical status classification (ASA class) [17], Eastern Cooperative Oncology Group performance status (ECOG PS) [18], comorbidities, cancer type, obstruction level, complications related to biliary drainage, cholangitis related to biliary obstruction or drainage, and laboratory values. The laboratory values were obtained within 7 days before PTBD and within 7 days after the diagnosis of cholangitis. The following laboratory values were analyzed: hemoglobin, leukocytes, thrombocytes, C-reactive protein (CRP), thromboplastin time international ratio (TT-INR), creatinine, albumin, bilirubin, alanine aminotransferase, and gamma glutamyl transferase. The blood culture and bile culture results after biliary drainage were also collected. The level of biliary obstruction was defined as upper (hilum), middle (common hepatic duct), or lower (common bile duct) based on the following radiological imaging techniques: ultrasound (US), computed tomography (CT), magnetic resonance imaging (MRI), or cholangiography. Infections were treated with antibiotics according to the current recommendations at our institution. Full electronic chemotherapy records were available between 2003 and 2016.

Time of death was acquired from death certificates (Statistics Finland) [19]. Survival was defined as the interval from PTBD to the patient’s death or last follow-up. The survival time for patients who received chemotherapy was defined as the time period from initiation of chemotherapy until patient death or last follow-up. The study protocol was approved by our institution’s ethics committee (No. 140/2011).

### Statistical analysis

All statistical analyses were performed using SPSS Statistics for Windows, Version 25.0 (IBM Corp, Armonk, NY). Summary data are presented as means with standard deviations (SDs) or medians with 25th and 75th percentiles. Between-group comparisons for continuous data were performed using analysis of variance (ANOVA) (> 2 groups) and Student’s t-test or Welch’s t-test when comparing two groups (the latter if the assumption of homoscedastic variances did not hold). Pearson’s χ^2^ test or Fisher’s exact test were used for categorical data. Log-rank tests were used to compare survival times in univariate analyses. A multivariable adjusted Cox proportional hazards model was used to determine the impact of cholangitis on 1-year mortality. To minimize the bias in the multivariable model, a directed acyclic graph (DAG) was used to create a minimally sufficient adjustment set. The DAG was drawn using the DAGitty tool [20]. The DAG model indicated that the following parameters should be taken into account in the Cox model: number of co-morbidities (none, 1 − 2, > 2), ASA class (1 − 2, 3, 4), type of cancer (pancreatic, biliary tract, metastatic gastrointestinal), the level of the biliary obstruction (upper, middle, lower), bleeding, and other complications. The results of the Cox model are presented as hazard ratios (HRs) and 95% confidence intervals (CIs). Two-tailed *P* values are reported.

## Results

In this series of 588 consecutive patients with gastrointestinal cancer and malignant biliary obstruction, 258 (43.9%) patients had pancreatic cancer, 222 (37.7%) had biliary tract cancer, and 108 (18.4%) had metastasis from cancers of the gastrointestinal tract. PTBD was performed instead of ERCP for the following reasons: Roux-en-Y reconstruction (n = 58, 9.9%), duodenal obstruction (n = 127, 21.6%), failed endoscopic drainage (n = 202, 34.4%), insufficient endoscopic drainage (n = 97, 16.5%), biliary obstruction in the hilum (n = 64, 10.9%), or some other reason (n = 40, 6.7%). A self-expanding metal stent (diameter 8 or 10 mm) was inserted in 455 patients (77.4%), a plastic stent (diameter 10 or 11.5 French) with combined ERCP was used in 18 patients (3.1%), and external drainage (diameter 8.5 or 10.2 French) was performed in 115 patients (19.5%).

Patient demographics and clinical characteristics are presented in Table 1: 156 patients (26.5%) had cholangitis before PTBD, 215 patients (36.6%) had cholangitis after PTBD, and 217 patients (36.9%) did not have cholangitis. The non-cholangitis group had fewer males than the groups with cholangitis (*P* = 0.005). Pancreatic cancer was the most common cancer among patients with cholangitis before PTBD (n = 69, 44.2%) and patients without cholangitis (n = 107, 49.3%, *P* = 0.043), whereas biliary tract cancer was most common among patients with cholangitis after PTBD (n = 98, 45.6%, *P* = 0.043). The obstruction was most often in the hilar area of the bile duct in cholangitis groups (41.7% and 49.8%), whereas in the non-cholangitis group the obstruction was most often in the lower level of the bile duct (42.9%, *P* = 0.029). There were more patients with ECOG PS 2 or higher in the cholangitis before PTBD group (*P* < 0.001), whereas the ASA class did not differ between the three groups (*P* = 0.16).

**Table 1:** Demographics and clinical characteristics of patients that underwent percutaneous transhepatic biliary drainage (PTBD) for malignant biliary obstruction.

		Onset of cholangitis		
	No cholangitis n = 217 (36.9%)	Before PTBD n = 156 (26.5%)	After PTBD n = 215 (36.6%)	*P* value^a^
Age, y; mean (SD [range])	71 (10 [40 − 91])	70 (11[38 − 90])	69 (12 [26 − 93])	0.097^b^
Sex, male	93 (42.9)	93 (59.6)	114 (53.0)	0.005
Comorbidities				0.26
0	68 (31.3)	46 (29.5)	58 (27.0)	
1–2	104 (47.9)	65 (41.7)	110 (51.2)	
> 2	45 (20.7)	45 (28.8)	47 (21.9)	
ECOG PS				< 0.001
0–1	100 (46.1)	45 (28.8)	116 (54.0)	
2	69 (31.8)	54 (34.6)	56 (26.0)	
3–4	48 (22.1)	57 (36.5)	43 (20.0)	
ASA class^c^				0.16
1–2	46 (21.6)	34 (22.2)	65 (30.7)	
3	143 (67.1)	99 (64.7)	129 (60.8)	
4	24 (11.3)	20 (13.1)	18 (8.5)	
Type of cancer				0.043
Pancreatic cancer	107 (49.3)	69 (44.2)	82 (38.1)	
Biliary tract cancer	70 (32.3)	54 (34.6)	98 (45.6)	
Metastatic gastrointestinal cancer	40 (18.4)	33 (21.2)	35 (16.3)	
Level of bile duct obstruction				0.029
Upper (hilum)	75 (34.6)	65 (41.7)	107 (49.8)	
Middle (common hepatic duct)	49 (22.6)	33 (21.2)	42 (19.5)	
Lower (common bile duct)	93 (42.9)	58 (37.2)	66 (30.7)	
Bilirubin level after PTBD <60.0 µmol/L^d^	74 (40.0)	67 (45.0)	80 (38.1)	0.42
Severity of cholangitis^e^				0.15
Mild		104 (66.7)	163 (75.8)	
Moderate		41 (26.3)	42 (19.5)	
Severe		11 (7.1)	10 (4.7)	
Complications of PTBD	62 (28.6)	72 (46.2)	105 (48.8)	< 0.001
Bleeding	53 (24.4)	58 (37.2)	87 (40.5)	< 0.001
Other^f^	8 (3.7)	20 (12.8)	21 (9.8)	0.004

Data are the number of patients (%), unless otherwise noted. ASA class, American Society of Anesthesiologists physical status classification; SD, standard deviation; ECOG PS, Eastern Cooperative Oncology Group performance status

^a^ Pearson chi-squared test, except for age

^b^ Analysis of variance test

^c^ Missing data for 10 patients

^d^ Missing data for 44 patients

^e^ According to Tokyo Guidelines 2013

^f^ Pleuritis, pneumonia, biliary or duodenal perforation, peritonitis or pancreatitis

Cholangitis was mostly mild in both cholangitis groups (Table 1). In the cholangitis before PTBD group, the median time from the onset of cholangitis to drainage was 5.5 days (25th − 75th percentiles 3.0 − 9.0) before the procedure. In the cholangitis after PTBD group, the median time from PTBD to cholangitis was 2.0 days (2.0 − 5.0). Patients without cholangitis had fewer complications related to PTBD than those with cholangitis (*P* < 0.001; Table 1). The most common complication was bleeding (n = 198, 33.7%); in most cases hemobilia. Most of the complications (n = 204, 85.4%) were treated conservatively, and relatively few patients (n = 35, 14.6%) were treated with interventional radiology or surgery. The 30-day mortality rate was 30.8% for patients with cholangitis before PTBD, 19.5% for patients with cholangitis after PTBD, and 25.8% for patients without cholangitis (*P* = 0.002; Table 2), while the 30-day mortality rate among patients without cholangitis or other PTBD-associated complications was 21.9%. The 30-day mortality rate was highest among patients with severe cholangitis (66.7%, n = 14; *P* < 0.001, Table 2).

**Table 2:** Outcome of patients with malignant biliary obstruction after percutaneous transhepatic biliary drainage (PTBD) according to the onset and severity of the cholangitis

		30-day mortality, n (%)	*P* value^a^	Median survival, months (95% CI)	1-year survival, n (%)	*P* value^a^
All, n = 588		146 (24.8)		2.7 (2.2 − 3.2)	95 (16.2)	
Onset of cholangitis			0.039			0.002
No cholangitis, n = 217, 36.9%		56 (25.8)		3.2 (2.4 − 4.1)	37 (17.1)	
Before PTBD, n = 156, 26.5%		48 (30.8)		1.8 (1.2 − 2.4)	14 (9.0)	
After PTBD, n = 215, 36.6%		42 (19.5)		3.0 (2.2 − 3.8)	44 (20.5)	
Severity of cholangitis^b^			< 0.001			< 0.001
Mild, n = 267, 72.0%		50 (18.7)		3.3 (2.5 − 4.0)	46 (17.2)	
Moderate, n = 83, 22.4%		26 (31.3)		1.9 (1.2 − 2.6)	11 (13.3)	
Severe, n = 21, 5.6%		14 (66.7)		0.3 (0.3 − 0.4)	1 (4.8)	

^a^ Log Rank

^b^ According to Tokyo Guidelines 2013

**Fig. 1:**
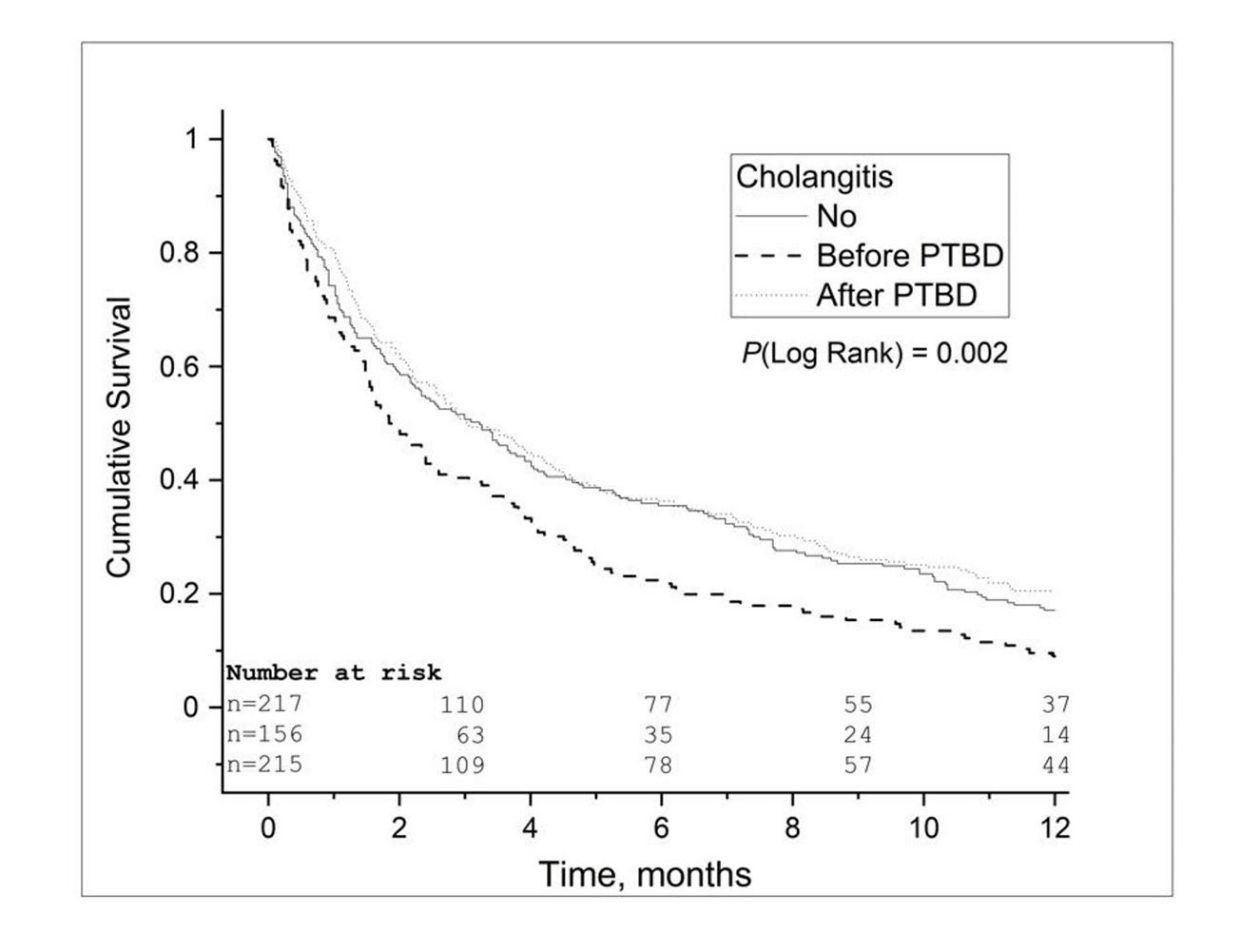
Kaplan-Meier 1-year survival analysis of patients that underwent percutaneous transhepatic biliary drainage (PTBD) for malignant biliary obstruction according to onset of cholangitis

**Fig. 2:**
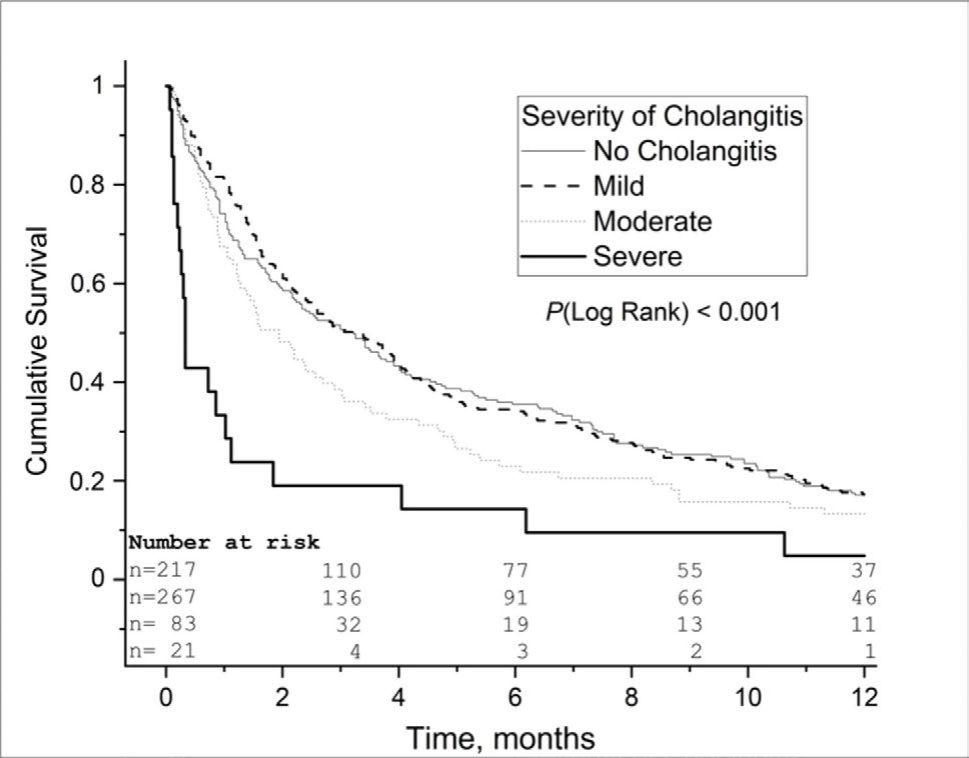
Kaplan-Meier 1-year survival analysis of patients that underwent percutaneous transhepatic biliary drainage (PTBD) for malignant biliary obstruction according to severity of cholangitis based on the Tokyo Guidelines 2013

Median survival was 1.8 months among patients with cholangitis before PTBD, 3.0 months among patients with cholangitis after PTBD, and 3.2 months among patients without cholangitis (*P* = 0.002; Table 2; Fig. 1). The median survival of the patients with mild cholangitis was 3.3 months, and 0.3 months for the patients with severe cholangitis (*P* < 0.001; Table 2; Fig. 2). One-year survival was 9.0% (n = 14) for the patients with cholangitis before PTBD, 20.5% (n = 44) for the patients with cholangitis after PTBD, and 17.1% (n = 37) for patients without cholangitis (*P* = 0.002; Table 2). In the multivariable adjusted Cox regression model, the HR for 1-year mortality for patients with cholangitis before PTBD was 1.3 (95% CI 1.06 − 1.67, *P* = 0.015) compared to the patients with cholangitis after PTBD. Furthermore, the HR for 1-year mortality was 1.4 (95% CI 0.99 − 1.95, *P* = 0.058) for patients with pancreatic cancer, 1.5 (95% CI 1.05–2.21, *P* = 0.026) with biliary tract cancer and 1.3 (95% CI 0.80–2.20, *P* = 0.27) for patients with metastatic gastrointestinal cancer, when the patients with cholangitis before PTBD compared to those with cholangitis after PTBD.

After successful PTBD, 54 out of 291 patients (18.6%) received chemotherapy: 10 (18.5%) in the group of cholangitis before the PTBD, 24 (44.4%) in the group with cholangitis after PTBD and 20 (37.0%) without cholangitis. After achieving chemotherapy, the median survival times were 5.2 months (95% CI 1.2–9.1) for patients with cholangitis before PTBD, 9.4 months (95% CI 3.9–15.0) for patients with cholangitis after PTBD and 15.3 months (95% CI 5.8–24.8) for patients without cholangitis (*P* = 0.12**)**.

## Discussion

Our result showed that the survival of patients with cholangitis before PTBD was poorer compared to patients with cholangitis after PTBD, whose survival did not differ statistically from patients without cholangitis. In all three groups, survival improved by several months if chemotherapy was received after successful biliary drainage.

In our study, the proportion of patients with cholangitis before PTBD was 26.5%, which is clearly higher than in earlier studies that varied from 7.2 to 11.3% [21–23]. The higher proportion was probably due to the fact that our tertiary level hospital offers PTBD services for the whole area of Northern Finland. Most of the earlier studies concerning cholangitis and PTPD have focused on hospital mortality and 30-day mortality. In a recent retrospective multicenter French study of 382 patients with acute cholangitis treated in intensive care units, the hospital mortality rate was 29%; obstruction non-related to gallstones and timing of drainage after 48 h were independent risk factors for mortality [24]. All our patients had malignant obstruction and the median delay before drainage was as much as 5.5 days. These two factors may explain our high 30-day mortality rate of 30.8%. Earlier biliary drainage would have probably improved survival in our series of patients. Patients with cholangitis prior to PTBD had poorer outcomes. In addition to delayed drainage, they also had poorer performance status than the other groups.

In our series, the incidence of cholangitis after PTBD was 49.8%, which is within the ranges of earlier studies from 8.4 to 66.2% [14, 21, 22, 25]. In interventional radiology, one dose of antimicrobial prophylaxis before a procedure including PTBD is recommended [26]. As far as we know there are three studies, including our present one, where antimicrobial prophylaxis was given only before the procedure. In our series, the incidence of early cholangitis within 24 h after drainage was 24.9%. The corresponding figure in a South Korean study was 25.9% [27]. We used cefuroxime prophylaxis and in the South Korean study the patients received cefotaxime. In a third study with a follow-up of up to 17 weeks [25], the incidence of cholangitis after PTBD was as high as 66.2%, but it should be noted that only external drainage was used. In that study, the antimicrobial prophylaxis given before the procedure was “a second-generation cephalosporin or according to the oncologist’s recommendation.“ On the other hand, there are two earlier studies, where the incidence of cholangitis after PTBD was clearly lower (11.1% and 15.7%) [13, 21]. In these studies, the duration of antimicrobial prophylaxis was three days after PTBD. So, it seems possible that one dose of prophylaxis before PTBD is not sufficient for prevention of cholangitis developing after the procedure for cancer patients with malignant biliary obstruction. Furthermore, in the abovementioned studies the used antimicrobial agents (cefoperazone sulbactam or ciprofloxacin) are more active against Gram-negative bacteria than cefuroxime.

The incidence of cholangitis after PTBD also seems to be dependent on the level of biliary obstruction. It has been shown that a stent across the duodenal papilla was the only independent predictor of therapeutic success [21]. In that study including three days of antimicrobial prophylaxis, the patients with a stent above the duodenal papilla had a significantly higher incidence of cholangitis (20%) than those patients who had a stent across the papilla (7%). In our series, 69% of the patients with cholangitis after PTBD had their biliary obstruction above the papilla. Also, in other studies, proximal biliary obstruction has been more challenging in terms of achieving complete drainage [28, 29], and is more often associated with infection [30, 31]. Taken together, one dose of antimicrobial prophylaxis and proximal obstruction are obvious explanations for our high incidence of cholangitis after PTBD.

Receiving chemotherapy after successful biliary drainage has a positive impact on survival after PTBD [6, 7, 32]. There are studies in which chemotherapy has been administered for 14–52% of gastrointestinal cancer patients after successful PTBD [21, 25, 33–35]. In one of these studies, the incidence of cholangitis was reported [25]. Although in that study chemotherapy after PTBD independently increased survival in the multivariate analysis, there were no direct comparisons of survival rates among patients receiving chemotherapy with and without cholangitis. In our real-life series about 19% of the patients received chemotherapy after successful biliary drainage with PTBD. The survival benefit with chemotherapy was 2.9- to 4.8-fold compared to patients without chemotherapy. The survival of patients with cholangitis before PTBD was poorer compared to patients with cholangitis after PTBD. Between cancer groups, this difference remained significant only for biliary tract cancer, but was no longer significant for pancreatic cancer or metastatic gastrointestinal cancer. However, the cancer-specific multivariable models would be more robust with a higher number of cases.

The main strength of our study was that we included all patients treated in our hospital with PTBD for malignant biliary obstruction due to the most common cancers causing biliary obstruction (pancreatic and biliary tract cancer) and also different types of metastatic gastrointestinal cancer, which we consider to be representative. PTBD procedures were performed by experienced interventional radiologists using previously documented methods and with the number of complications in line with previous studies [11, 12, 36]. A directed acyclic graph (DAG) was used to examine biases related to possible causal variables and to create a minimally sufficient adjustment set to avoid misleading conclusions [20, 37]. In our series of malignant biliary obstruction, the Tokyo Guidelines proved to be applicable in grading the severity of cholangitis and predicting patient outcomes, although the use of the Tokyo Guidelines in association with malignant biliary obstruction has been questioned earlier [23]. The main limitation of our study was that it was a single-center retrospective study, like many of the previous studies on this subject. Unfortunately, our retrospective study did not allow a precise evaluation the causes of cholangitis, such as blockage or slippage of PTBD catheter or inadequately drained system, after PTBD.

## Conclusion

For gastrointestinal cancers with malignant biliary obstruction, survival is poorer if cholangitis occurs before PTBD than if cholangitis occurs after PTBD. In these patients an early biliary drainage and antimicrobial treatment is required to improve survival. For preventing cholangitis after PTBD, more than one dose of antimicrobial prophylaxis seems to be reasonable. Due to the notable survival benefit, consultation with an oncologist to assess the possibility of chemotherapy is essential in successfully treated PTBD patients.

## Data Availability

Finnish legislation prohibits the distribution or otherwise making patient data directly available to other parties. Thus, while the authors have the required authorizations to access the data, it may not be shared with or forwarded to other researchers, nor is it publicly available. The corresponding author can be contacted for data availability.

## Abbreviations

ANOVA: Analysis of variance
ASA: class American Society of Anesthesiologists physical status classification
CI: Confidence intervals
CRP: C-reactive protein
CT: Computed tomography
DAG: Directed acyclic graph
ECOG: PS Eastern Cooperative Oncology Group performance status
ERCP: Endoscopic retrograde cholangiopancreatography
EUS: Endoscopic ultrasound
EUS-BD: Endoscopic ultrasound-guided biliary drainage
HR: Hazard ratio
MRI: Magnetic resonance imaging
PTBD: Percutaneous transhepatic biliary drainage
SD: Standard deviation
TG13: Tokyo Guidelines 2013
TT-INR: Thromboplastin time international ratio
US: Ultrasound
